# Modification of Pillared Intercalated Montmorillonite Clay as Heterogeneous Pd Catalyst Supports

**DOI:** 10.3390/molecules28227638

**Published:** 2023-11-17

**Authors:** Kailang Sun, Yonghong Liu, Taojun Zhang, Jie Zhou, Jinyang Chen, Xiaorong Ren, Zhen Yang, Minfeng Zeng

**Affiliations:** Research Center of Advanced Catalytic Materials & Functional Molecular Synthesis, Zhejiang Key Laboratory of Alternative Technologies for Fine Chemicals Process, College of Chemistry & Chemical Engineering, Shaoxing University, Shaoxing 312000, China; sunklfei@163.com (K.S.); xiangchiyaoguai@163.com (Y.L.); ztjbuhejiu@sina.com (T.Z.); zjbuchouyan@126.com (J.Z.); 2023000069@usx.edu.cn (J.C.); renxiaorong@usx.edu.cn (X.R.); yangzhen09@usx.edu.cn (Z.Y.)

**Keywords:** montmorillonite clay, pillaring, heterogeneous catalyst

## Abstract

Montmorillonite clay was modified by pillaring with AlMn oxides in different Al/Mn ratios and intercalation of two kinds of N-containing polymers (i.e., chitosan (CS) and polyvinyl pyrrolidinone (PVP)) chains. The modified pillared montmorillonite clay (PM) showed a rich two-dimensional layered porous structure with tunable parameters, such as large interlayer spacing, high specific area, and large porous volume. They were then used as supports for Pd nanoparticles. As applied in coupling reactions of aryl halides with terminal alkynes, Pd@CS/AlMn-PM showed better comprehensive catalytic performance than Pd@PVP/AlMn-PM. This was mainly attributed to its higher specific area, stronger chelation to Pd species, and better solvent resistance.

## 1. Introduction

By utilizing transition metal, especially palladium (Pd) nanoparticles catalyzed cross coupling reaction technology, people can accurately and efficiently construct carbon-carbon, carbon-hetero, and other new chemical bonds to produce various complex organic compounds [1,2,3]. In recent decades, it has been widely used in various fields of organic synthesis chemistry, advanced functional materials, biopharmaceuticals, and so on. In traditional homogeneous catalytic systems, active Pd catalysts are often directly put into the reaction system as catalysts. However, the precious Pd metal species is difficult to separate and recycle, suffering the drawbacks of being uneconomical and unfriendly to the environment. Immobilizing Pd catalysts onto inert or functional solid materials to produce a supported heterogeneous palladium catalyst is a good solution to release these drawbacks. In heterogeneous catalysis processes, the reactions can be also effectively catalyzed. Moreover, the supported Pd catalysts can be easily separated after the reaction and conveniently reused in a next reaction cycle. Therefore, the development of high-performance heterogeneous palladium catalysts immobilized on suitable supports has received increasing attention in both academic and industrial fields [4,5,6].

Recently, using natural silicate clay mineral as support for active metal nanoparticles to prepare a variety of reusable functional solid materials with high catalytic performance as catalysts for green chemical processes has become a more and more attractive field [7,8,9]. The structural unit layer of natural silicate clay mineral is usually composed of Si-O tetrahedral sheets and Al-O octahedral sheets. According to their ratio, it can be divided into 1:1 type kaolinite clay and 2:1 type montmorillonite clay, respectively. Through strong covalent bonding, Si-O tetrahedral sheets and Al-O octahedral sheets are well connected to each other. However, other metal ions with different valence states are prone to isomorphic substitution with the central atoms in octahedral sheets (such as Mg replacing Al) or tetrahedral sheets (such as Al replacing Si), resulting in negative charges on the layer. Positively charged cations (usually Na+ and Ca^2+^) exist in the interlayer to compensate for the negative charges carried in the layer, and the layers are bonded together through electrostatic and van der Waals forces, thus forming gaps between the layers. The distance between the clay layers can be effectively enlarged by means of shear stirring, intercalation treatment, metal oxides pillaring, and so on. For example, pillared intercalated montmorillonite clays (PM) can be prepared by intercalation of inorganic or organic compounds between the clay layers, resulting in an increased basal spacing, enlarged interlayer spaces, larger pore volume, and higher surface specific area. Due to such textural structure characteristics, PM-based materials are highly attractive for adsorption and catalytic applications [10,11,12]. For example, active Pd nanoparticles are readily introduced into the interlayer spaces of PM to prepare novel advanced catalytic composites for combustion of volatile organic compounds, combustion of propene, and C-C coupling reactions [13,14,15,16].

One of the most frequently used inorganic pillaring agents is multivalent polyhy-droxyl-Al^3+^ cations [17,18]. The polycations can be effectively intercalated into the interlayer spaces of the clay mineral by substituting the exchangeable charge-compensating cations. Followed by dehydration and dihydroxylation, stable Al oxide pillars will form between the layers with permanently enlarged interlayer spaces, which is so-called Al-PM. The interlayer spacing, pore volume, specific surface area, and thermal stability of the Al-PM can be further improved by introducing mixed-oxide pillars, such as AlFe, AlCu, AlSi, AlTi, AlGa, AlZr, AlNi, etc. [19,20,21]. The study of the preparation of AlMn–PMs has been rather scarce regarding its lower doping efficiency with Al oxides pillars. In addition, Mn and its compounds have been widely used as catalytic species for many organic reactions in homogeneous phase [19].

In this study, an Mn species was successfully incorporated to Al pillaring agents with different mol ratios to prepare novel double-core AlMn-PM with tunable texture structure characteristics. Moreover, the affinity to organic molecules of the PM layers was further improved by intercalation treatment with two kinds of N-containing polymers, such as chitosan (CS) and polyvinyl pyrrolidone (PVP). Both polymers have high affinity to clay-based materials. Meanwhile, they have been proven to be a good stabilizer for Pd species. The prepared CS/AlMn-PM or PVP/AlMn-PM has been used as the carrier for Pd nanoparticles to prepare novel Pd@CS/AlMn-PM or PVP/AlMn-PM. The catalytic performances of the novel catalysts for Sonogashira reactions of aryl halides with terminal alkynes have been comparably evaluated. 

## 2. Results and Discussions

### Microstructure of the Catalytic Nanocomposites

**Figure 1:**
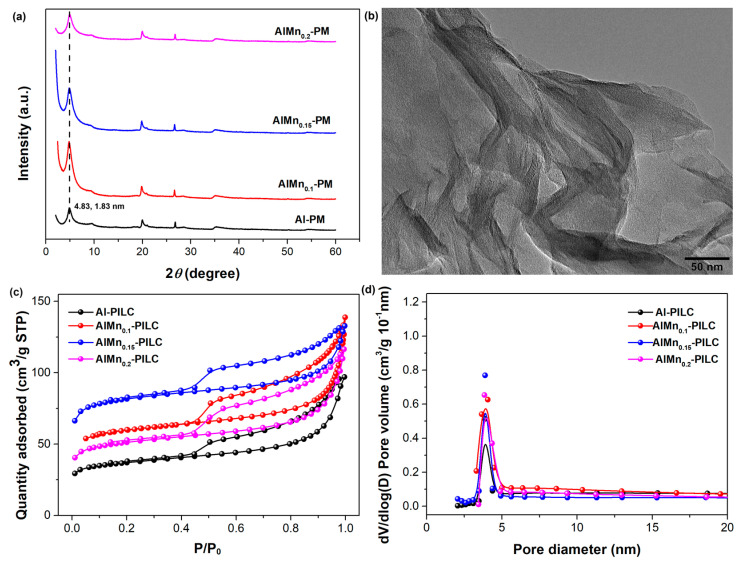
Microstructure characterization results of the AlMn-PM: (**a**) XRD patterns; (**b**). HRTEM observation of AlMn_0.15_-PM; (**c**) N_2_ adsorption–desorption isotherm curves; (**d**) pore distribution.

The effect of the Mn doping ratio on the AlMn-PM was evaluated first. The volume ratios of the Al^3+^/Mn^2+^ with the same concentration of 0.2 mol/L were set as 1/0, 1/0.1, 1/0.15, and 1/0.2, respectively. As shown in Figure 1a, the characteristic (001) diffraction peaks are located at 2*θ* of 4.83° for all the samples, indicating the doping of Mn has little effect on the basal spacing of the silicate layers. And the basal spacing can be calculated as about 1.83 nm with the famous Bragg equation, which is much larger than the starting montmorillonite clay of about 1.25 nm, confirming the successful pillaring modification by Al or AlMn. The pillared intercalated clay shows distinct well-ordered layer-stacking morphology as observed from HRTEM (Figure 1b). The porous structure of the AlMn-PM has been characterized by N_2_ adsorption–desorption isotherms. As shown in Figure 1c, the isotherms of all the samples can be classified as type IV curves with typical H4-type hysteresis loops at higher *P*/*P*_0_ (0.45), indicating rich layered mesoporous structure. As shown in Figure 1d, all the samples show similar pore size distribution range with a peak at about 3.9 nm. The structure parameters of total surface area (*S*_BET_), pore volume (*V*_p_), and mesoporous area (*V*_mes_) of the samples were extracted from the isotherm curves, and the results were shown in Table 1. The Al-PM shows *S*_BET_ of 126.59 m^2^/g, *V*_tot_ of 0.13 cm^3^/g, and *V*_meso_ of 0.11 cm^3^/g, respectively. After being doped with Mn, the *S*_BET_ of the AlMn-PM shows an effective increase to 186.88 m^2^/g (AlMn_0.1_-PM), 277.53 m^2^/g (AlMn_0.15_-PM), and 178.43 m^2^/g (AlMn_0.2_-PM), respectively. As does the total pore volume of the AlMn-PM. This means that the doping of Mn is in favor of the improvement of the adsorption properties of the AlMn-PM. Meanwhile, for all the samples, *V*_meso_/*V*_tot_ is higher than 2/3, confirming the high percentage of the mesoporous structure derived. It is hopeful that the AlMn-PM with high adsorption properties should be good candidate support for Pd metal nanoparticles catalysts. Clearly, the best modification effects are observed in the case of AlMn0.15-PM, which shows highest *S*_BET_ and *V*_tot_. Therefore, AlMn_0.15_-PM has been selected as the support candidate for the further development of novel heterogeneous catalysts.

**Figure 2:**
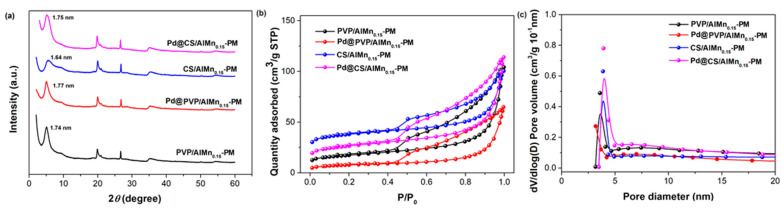
Microstructure characterization results of the Pd@polymer/AlMn_0.15_-PM nanocomposites: (**a**) XRD patterns; (**b**) N_2_ adsorption–desorption isotherm curves; (**c**). pore distribution.

Polymer chains and Pd nanoparticles were further introduced to the porous AlMn-PM matrix. The corresponding effects on the microstructure changes of the polymer/AlMn-PM supports and Pd@polymer/AlMn_0.15_-PM were tracked by XRD, HRTEM, and N_2_ adsorption–desorption characterizations. As shown in Figure 2a, the introduction of polymer chains leads to a slight decrease in basal spacing to 1.74 nm (PVP/AlMn_0.15_-PM) and 1.64 nm (CS/AlMn_0.15_-PM), respectively. This should be attributed to some interference of polymer chains on the formation of perfect inorganic pillars. Meanwhile, it was found that the basal spacing underwent a slight increase to 1.77 nm (Pd@PVP/AlMn_0.15_-PM) and 1.75 nm (Pd@CS/AlMn_0.15_-PM) after further immobilization of Pd nanoparticles. This might be attributed to some pillaring effects contributed by the Pd nanoparticles which are clipped in the interlayer spaces.

As shown in Figure 2b,c, though the pore size distribution undergoes little change, the adsorption capability decreases obviously after both polymer chains and Pd species loading. As shown in Table 2, *S*_BET_ decreases obviously to 60.49 m^2^/g (PVP/AlMn_0.15_-PM), 27.66 m^2^/g (Pd@PVP/AlMn_0.15_-PM), 129.76 m^2^/g (CS/AlMn_0.15_-PM), and 91.55 m^2^/g (Pd@CS/AlMn_0.15_-PM), respectively. And so do the total pore volume (*V*_tot_), such as 0.13 cm^3^/g (PVP/AlMn_0.15-_PM), 0.09 cm^3^/g (PVP/AlMn_0.15_-PM), 0.14 cm^3^/g (CS/AlMn_0.15_-PM), and 0.13 cm^3^/g (Pd@CS/AlMn_0.15_-PM), respectively. The intercalated polymer chains and loaded Pd nanoparticles have a blocking effect on the layered mesopores of the nano-composite. Clearly, Pd@CS/AlMn_0.15_-PM has an obviously larger surface area and porous volume than Pd@PVP/AlMn_0.15_-PM. This might be mainly attributed to the higher compatibility between the CS chains and PM matrix. As we know, -OH and -NH_2_ groups (within CS chains) will reasonably form a stronger interaction with Si-OH groups of PM matrix than amide N-C=O groups (within PVP chains). Nevertheless, the derived Pd@polymer/AlMn_0.15_-PM catalysts show large specific surface areas comparable with other recent prepared Pd heterogeneous catalysts supported on clay or polymer-based supports [22,23,24,25].

**Figure 3:**
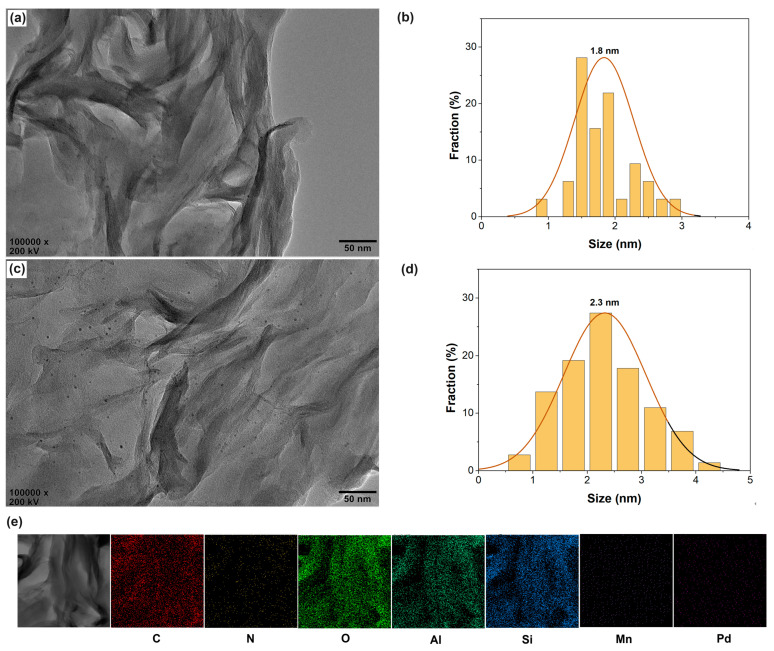
HRTEM observation of the Pd@polymer/AlMn_0.15_-PM nanocomposites: (**a**) Pd@CS/AlMn_0.15_-PM; (**b**) Pd nanoparticles size distribution of Pd@CS/AlMn_0.15_-PM; (**c**) Pd@PVP/AlMn_0.15_-PM; (**d**) Pd nanoparticles size distribution of Pd@PVP/AlMn_0.15_-PM; (**e**) EDX mapping results of Pd@CS/AlMn_0.15_-PM.

The phase behavior was further studied by the HRTEM characterization. As shown in Figure 3a,b, nanosized Pd particles (average size of about 1.8 nm) disperse well, mainly in the interlayer spaces of CS/AlMn_0.15_-PM supports. Pd@PVP/AlMn_0.15_-PM shows similar phase behavior but a bit larger in size of dispersed Pd nanoparticles (average size of about 2.3 nm). The HRTEM EDX-mapping results confirm the presence of the elements, such as C, N, O, Al, Si, Mn, and Pd, respectively (as shown in Figure 3e). In summary, these phenomena indicate that both CS chains and PVP chains have been successfully encapsulated in the interlayer spaces of the PM matrix. And CS chains show a bit of a higher promotion effect on the nanosized Pd species dispersion in the interlayer spaces of PM matrix than that of PVP chains. This should be attributed to the stronger chelation of CS chains to Pd species.

**Figure 4:**
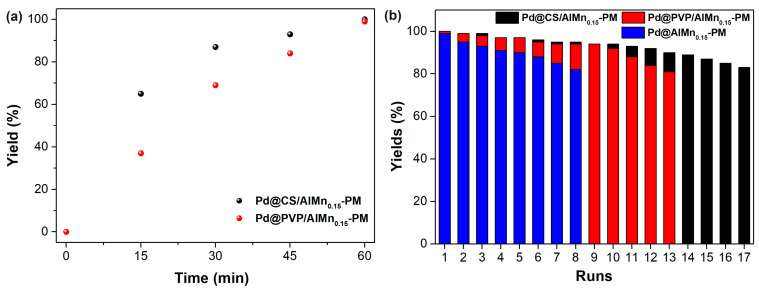
Sognogashira reaction yield vs reaction time (**a**) and recycling runs (**b**) as catalyzed by the prepared catalysts.

The catalytic performances of the prepared Pd@polymer/AlMn_0.15_-PM nanocomposite were evaluated with a Sonogashira reaction of aryl halides and terminal alkynes. Iodo benzene coupling with phenylacetylene was selected as model reaction. The appropriate reaction conditions were adopted from our former works, such as DMSO as solvent, ethylene glycol as reductive additives, and a reaction temperature of 90 °C. Under the optimized reaction conditions, the activity of the catalytic nanocomposites was monitored vs the reaction time. As shown in Figure 4a, as catalyzed by Pd@CS/AlMn_0.15_-PM, the model reaction yields reached 65% (15 min), 87% (30 min), 93% (45 min), and 100% (60 min), respectively, exhibiting excellent catalytic efficiency. For comparison, the corresponding yields after same reaction time as catalyzed by Pd@CS/Al-PM were seen as 37% (15 min), 69% (30 min), 84% (45 min), and 99% (60 min), respectively. Clearly, Pd@CS/AlMn_0.15_-PM shows better catalytic efficiency than Pd@PVP/AlMn_0.15_-PM. The Pd@CS/AlMn_0.15_-PM had higher adsorption performances than Pd@PVP/AlMn_0.15_-PM, which was confirmed in the above N_2_ adsorption–desorption characterization results. In addition, Pd nanoparticles with a smaller size in the case of Pd@CS/AlMn_0.15_-PM is beneficial for better catalytic activity performances. The Pd@polymer/AlMn_0.15_-PM nanocomposites are convenient for separation from the reaction medium, and reused in next reaction run. Figure 4b shows the relationship between the yield of the reaction and recycling runs of the catalytic materials, such as Pd@CS/AlMn_0.15_-PM, Pd@PVP/AlMn_0.15_-PM, and Pd@AlMn_0.15_-PM. To maintain a coupling yield higher than 80%, the nanocomposite can be reused for 17 runs (Pd@CS/AlMn_0.15_-PM), 13 runs (Pd@PVP/AlMn_0.15_-PM), or 8 runs (Pd@AlMn_0.15_-PM). Overall, although the intercalated polymer chains will sacrifice the surface area of the catalyst, the encapsulation of the N-containing polymer chains, which can form strong chelation to the Pd nanoparticles, is beneficial for the improvement of the recycling stability of the heterogeneous catalyst. In addition, PVP chains have good solubility in polar solvents [26,27]. The reason for the better performances of the Pd@CS/AlMn_0.15_-PM catalyst should be attributed to its stronger chelation with Pd species and better solvent resistance. 

Besides the model reaction, the Pd@polymer/AlMn_0.15_-PM nanocomposite catalysis system can be well extended to the Sonogashira coupling reactions of other aryl halides with phenyl acetylene. As shown in Table 3, both Pd@CS/AlMn_0.15_-PM and Pd@PVP/AlMn_0.15_-PM nanocomposite catalyst systems show a similar high catalytic efficiency for substituted iodo benzene, whether having electron-donating groups (entries 2, 3) electron-withdrawing groups (entries 4, 5) or a different substitution position. Meanwhile, the catalytic system also shows high catalytic efficiency for phenylacetylene substituted with both electron-donating group (entries 6, 7) and electron-withdrawing group (entries 8, 9). It also shows good catalytic efficiency for the Sonogashira reaction of aryl bromides with terminal alkynes. The coupling yield of bromo benzene with phenylacetylene is 35% (entry 10), due to the higher bonding energy of C-Br than C-I. Substitution with electron-withdrawing groups can further activate the C-Br bonding with obviously higher yields (entries 13–15). Whereas, electron-donating groups have an opposite effect on the breaking of C-Br bonding, resulting in low yield (entry 11, 12). For a similar reaction with similar reaction conditions, the novel catalysts prepared in this work show superior comprehensive catalytic performances to other recent Pd heterogeneous catalytic systems [28,29,30,31].

## 3. Materials and Methods

### 3.1. Materials

Montmorillonite (Na^+^ type clay) was supplied by Nanocor Co., Arlington Heights, IL, USA. Chitosan (molecular weight of 1.2 × 10^5^ and deacetylated degree of 95%) was supplied by Yuhuan, Zhejiang Aoxing Biotechnology Co., Ltd., Zhejiang, China. Polyvinyl pyrrolidinone (K-30 type, molecular weight of 4 × 10^4^) was supplied by Sinopharm Chemical Reagent Co., Ltd., Shanghai, China. PdCl_2_ salt was supplied by Zhejiang Metallurgical Research Institute Co., Ltd., Hangzhou, Zhejiang, China. Multivalent metal source of AlCl_3_⋅6H_2_O, Mn(NO_3_)_2_⋅6H_2_O and other reagents were supplied by Sinopharm Chemical Reagent Co., Ltd., Shanghai, China. Aryl halides and terminal alkynes reactants involved in Sonogashira reactions were supplied by Energy Chemical, Sun Chemical Technology Co., Ltd., Shanghai, China. 

### 3.2. Catalyst Preparation

The preparation process of the AlMn-PM nanocomposite is in accordance with recent work of ref [32]. The AlMn oxide pillar sources used were 0.2 mol/L of AlCl_3_ and Mn(NO_3_)_2_ solution. The mixing volume ratios AlCl_3_ and Mn(NO_3_)_2_ solution were set as 1/0, 1/0.1, 1/0.15, and 1/0.2, respectively. A certain amount of 0.4 mol/L of NaOH solution was dropwise added to the mixed solution until the mol ratio of (OH^−^)/(Al^3+^-Mn^2+^) reached 2.4, at a condition of 60 °C heating and stirring for 6 h. Then, the mixture was aged at 60 °C for 12 h to obtain AlMn pillaring agent. The pillaring agent was dropwise added into 10 wt% montmorillonite clay suspension according the mass ratio of (20 mmol of polyhydroxy metal cations/1 g of clay) to obtain AlMn_x_-PM (x = 0.15) precursor at a condition of 60 °C heating and stirring for 6 h. According to the polymer/clay mass ratio of 1/9, a certain amount of 2wt % CS or PVP acetic solution was dropwise added to the AlMn_0.15_-PM precursor and stirred at 60 °C for 2 h. Then, 2 mL of 0.3wt% PdCl_2_ solution (dissolved with the presence of NaCl) was dropwise added into the polymer/AlMn_0.15_-PM precursor composite suspension and stirred at 60 °C for 0.5 h. The Pd@polymer/AlMn_0.15_-PM precursor was centrifuged and washed with deionized water to neutralise without Cl^−^. After drying, polymer/AlMn_0.15_-PM supports or Pd@polymer/AlMn_0.15_-PM precursor was heat treated in a tubular muffle furnace (BTF-1600C, Anhui BEQ Equipment Technology Co., Ltd., Hefei, China) at moderate temperature of 200 °C (in N_2_ atmosphere) for 12 h to obtain the final Pd supported catalysts. The actual polymer content within the heterogeneous catalysts was measured by thermogravimetric analysis (TGA). PVP and CS within the Pd@polymer/AlMn_0.15_-PM was determined as 6.3%, 9.2%, respectively. 

### 3.3. Catalyst Characterization

The N_2_ adsorption experiments were performed using a TriStar II 3020 apparatus (Micromeritics Company, Norcross, GA, USA) at −196 °C of liquid N_2_ temperature. All samples were degassed at 200 °C for 4 h at a pressure lower than 0.133 Pa. The BET specific surface area (*S*_BET_) was calculated from the nitrogen adsorption vs relative pressure curve in the range of 0.1–0.5 with the Brumanuer–Emmet–Teller method. The total volume of pores (*V*_p_) was estimated from the N_2_ quantity absorbed at a relative pressure of *P*/*P*_0_ = 0.99. The mesoporous volume (*V*_meso_) was estimated using the BJH method. The X-ray diffraction experiments were performed using the Empyrean diffraction system (PANalytical company, Netherlands), at scanning rate of 2 °/min in the 2*θ* range of 2 to 60°. Thermogravimetric analysis (TGA) of the samples was performed with a Mettler Toledo TGA/DSC 2 STARe system (Columbus, OH, USA), with speed of 20 °C/min in air atmosphere. Morphology observation of the samples was performed using a JEM-2100 F high resolution transmission electron microscope HR-TEM (JEOL Ltd., Tokyo, Japan), equipped with an energy dispersive X-ray spectroscopy (EDX, Oxford EDX System, Oxfordshire, UK). X-ray photoelectron spectroscopy (XPS) analysis of the samples was performed using a Thermo Scientific ESCALAB 250Xi spectrometer (Waltham, MA, USA). The determination of Pd contents was performed using a Leemann ICP-AES Prodigy XP inductively coupled plasma-atomic emission spectrometer (Hudson, NH, USA). The samples were pretreated with the mixed solution of concentrated HCl/fuming HNO_3_ (3/1), and then diluted. The Pd content within Pd@CS/AlMn_0.15_-PM and Pd@PVP/AlMn_0.15_-PM was determined as 1.7% and 1.4%, respectively.

### 3.4. Catalyst Performances

Sonogashira coupling reactions of aryl halides and terminal alkynes were carried out as follows. In a 50 mL reaction tube, a mixture of 1 mmol of aryl halides reactants, 1.2 mmol of terminal alkynes reactants, heterogenous Pd catalysts (containing 2.3 μmol of Pd), 3 mmol of CH_3_COOK, 0.2 mL of ethylene glycol, and 5 mL of solvents was added and magnetically stirred at 90 °C (heated in an oil bath) for 1 h. The structure of the reaction products was confirmed with ^1^HNMR spectra using Brucker400-Hz NMR and mass spectra using Agilent 6890N/5975 MSD GC/MS. The yields of the reaction were measured by GC/MS quantitative analysis based on peak-area of the coupling products and unreacted aryl halide reactant using normalization method. All the structure information was in good agreement with our recent works [16,33,34]. The recycling experiments of Pd heterogeneous catalysts were performed using the model coupling reaction of iodo benzene and phenyl acetylene. The catalysts were filtrated out the reaction system after each run. Then they were washed with ethanol and dried. Finally, they were added into the next reaction.

## 4. Conclusions

The layered mesoporous structure of PM can be tuned by the modification of AlMn pillaring, resulting in effective improving of the adsorption capabilities, such as higher specific surface area and pore volumes. Further incorporation of polar polymers can further improve the chelation capability with Pd nanoparticle species. The resultant Pd@polymer/AlMn_0.15_-PM heterogenous catalytic nanocomposites shows highly competitive catalytic performances as used for Sonogashira reactions. Pd@CS/AlMn-PM shows better comprehensive catalytic performance than Pd@PVP/AlMn-PM, which should be mainly attributed to its higher specific area, stronger chelation to Pd species, and better solvent resistance. Undoubtedly, this work enriches the catalytic functional materials starting from nature-rich sources of clay, and supplies a new choice of heterogeneous transition metal catalyst for the clean production process of fine chemical synthesis.

## Figures and Tables

**Table 1:** Structure parameter of AlMn-PM with different nanocomposites AlMn ratio extracted from the isotherms in Figure 1c.

Samples	*S*_BET_ (m^2^/g)	*V*_tot_ (cm^3^/g)	*V*_meso_ (cm^3^/g)
Al-PM	126.59	0.13	0.11
AlMn_0.1_-PM	186.88	0.18	0.13
AlMn_0.15_-PM	277.53	0.18	0.12
AlMn_0.2_-PM	178.43	0.15	0.10

*S*_BET_: specific surface area, by Brumanuer-Emmet-Teller method; *V*_tot_: total volume of pores, the N_2_ quantity absorbed at a relative pressure of *P*/*P*^0^ = 0.99; *V*_meso_: volume of mesopores obtained by BJH method.

**Table 2:** Structure parameter of CS/AlMn-PM support and Pd@CS/AlMn-PM nanocomposites extracted from the isotherms in Figure 2b.

Samples	*S*_BET_ (m^2^/g)	*V*_tot_ (cm^3^/g)	*V*_meso_ (cm^3^/g)
PVP/AlMn_0.15_-PM	60.49	0.13	0.11
Pd@PVP/AlMn_0.15_-PM	27.66	0.09	0.07
CS/AlMn_0.15_-PM	129.76	0.14	0.13
Pd@CS/AlMn_0.15_-PM	91.55	0.13	0.12

**Table 3:** Sonogashira coupling reactions of various aryl halides with terminal alkynes catalyzed by Pd@CS/AlMn_0.15_-PM (C1) and Pd@PVP/AlMn_0.15_-PM (C2) catalytic materials.

Entry	Aromatic Halides	Alkynes	Yield ^a^ (%)	
			C1	C2
1	C_6_H_5_I	C_6_H_5_C≡CH	100	99
2	3-CH_3_C_6_H_4_I	C_6_H_5_C≡CH	99	95
3	4-OCH_3_C_6_H_4_I	C_6_H_5_C≡CH	98	94
4	4-ClC_6_H_4_I	C_6_H_5_C≡CH	96	92
5	3-COCH_3_C_6_H_4_I	C_6_H_5_C≡CH	98	93
6	C_6_H_5_I	4-CH_3_C_6_H_4_C≡CH	97	97
7	C_6_H_5_I	4-OCH_3_C_6_H_4_C≡CH	92	89
8	C_6_H_5_I	4-ClC_6_H_4_C≡CH	95	91
9	C_6_H_5_I	4-BrC_6_H_4_C≡CH	90	90
10	C_6_H_5_Br	C_6_H_5_C≡CH	35 ^b^	35 ^b^
11	4-CH_3_C_6_H_4_Br	C_6_H_5_C≡CH	23 ^b^	25 ^b^
12	3-OCH_3_C_6_H_4_Br	C_6_H_5_C≡CH	21 ^b^	20 ^b^
13	3-COCH_3_C_6_H_4_Br	C_6_H_5_C≡CH	72 ^b^	70 ^b^
14	4-COCH_3_C_6_H_4_Br	C_6_H_5_C≡CH	82 ^b^	80 ^b^
15	4-ClC_6_H_4_Br	C_6_H_5_C≡CH	78 ^b^	71 ^b^

^a^ GC-MS yield, ^b^ the reaction time is 3 h, and the rest reaction time is 1 h.

## Data Availability

Data are available from authors based on reasonable requirement from readers.
